# Hepatitis C among healthcare personnel: secondary data analyses of costs and trends for hepatitis C infections with occupational causes

**DOI:** 10.1186/s12995-016-0142-5

**Published:** 2016-11-24

**Authors:** Claudia Westermann, Madeleine Dulon, Dana Wendeler, Albert Nienhaus

**Affiliations:** 1 University Medical Center Hamburg-Eppendorf (UKE), Center of Excellence for Health Services Research in Nursing (CVcare), Hamburg, Germany; 2 Institution for Statutory Accident Insurance and Prevention in the Health and Welfare Services (BGW), Department of Prevention and Rehabilitation Principles (GPR), Hamburg, Germany

**Keywords:** Hepatitis C virus, Occupational disease, Healthcare personnel, Burden of disease, Secondary data analysis

## Abstract

**Background:**

Hepatitis C infection is a global public health issue. Chronic hepatitis C infection is associated with significant morbidity and mortality. The aim of this study is to describe the costs for occupationally-cased hepatitis C infections based on data from an accident insurance carrier.

**Methods:**

This study is a secondary analysis based on the Database of a German Institution for Statutory Accident Insurance. The analysis is based on a sample of insured parties whose hepatitis C infections were recorded as occupational diseases between 1996 and 2013. The analysis is based on recognised hepatitis C cases and incorporates records registered between 1 January 2000 and 31 December 2014.

**Results:**

Within the study period, the number of reported and recognised hepatitis C cases declined by 73 and 86% respectively. The majority of recognised hepatitis C cases (*n* = 1.121) were female, older than 40 years and were active in a medical nursing profession. In the study period, the costs came to a total of € 87.9 million, of which 60% was attributable to pension payments (€ 51,570,830) and around 15% was attributable to pharmaceutical and medicinal products (€ 12,978,318). Expenses for drugs exhibited heavy increases in 2012 (from around € 500,000–800,000 to € 1.7 million) and 2014 (to € 2.5 million) in particular. Pension payments came to € 1.6 million in 2000 and rose continuously to over € 4 million in 2014. Expenses for occupational rehabilitation accounted for less than 1%.

**Conclusions:**

For hepatitis C infections as an occupational disease, a considerable increase in costs has been observed in recent years, while the number of reports has declined heavily. This rise in costs is explained by the increase in pension payments and, since 2012, by a rise in the costs for drugs. The high costs of anti-viral therapies is offset by the potential for considerable treatment benefits. Healing the infection is expected to generate long-term cost savings for statutory accident insurance carriers, and also for social security systems.

## Background

The viral disease hepatitis C (HCV) is globally one of the most common infectious diseases. The hepatitis C RNA virus is transmitted from person to person, primarily through contact with infected blood while the skin or mucosa is also injured [1]. Healthcare personnel are at greater risk of HCV infection due to the nature of their occupational duties [2]. The progression of the infection is often non-specific, which is why the infection often remains undetected. Up to 85% of infections are chronic (HCV-RNA positive longer than six months). According to data from the World Health Organisation (WHO), around 150 million people globally have chronic hepatitis C (CHC); 700,000 people die each year as a result [1, 3, 4]. CHC is associated with a high level of morbidity and a diminished quality of life. Up to 68% of patients suffer from symptoms including fatigue, exhaustion, limited performance and sub-clinical cognitive impairment. CHC is also associated with an increased risk for developing cirrhosis of the liver and hepatocellular carcinoma [4, 5]. A variety of extrahepatic manifestations may also develop. The development of depression symptoms, diabetes mellitus and malignant lymphoproliferative disorders such as follicular non-Hodgkin lymphoma are associated with HCV. Several studies have also indicated impairment of certain central nervous functions and neurotransmission [4]. US scientists have calculated the costs for the US healthcare system associated with CHC on the basis of data from a private health insurance company. They (retrospectively) studied the insurance data of 53,796 CHC patients over a period of eight years (2002–2010), according to which the average cost attributable to CHC per patient, per year was $ 24,176 (approx. € 21,776). Stratified according to stages of the disease, these costs increase as the disease progresses, reaching their peak in patients with terminal cirrhosis of the liver ($ 59,995/€ 53,880 per patient, per year) [6]. According to estimates, the share of patients with manifest liver disease will quadruple over the next 20 years without effective treatment [7]. Due to the potentially severe progression of the disease and the high associated costs, successful treatment of CHC is important [3, 6, 7]. The use of second-generation direct anti-viral agents (DAAs) today provide promising treatment combinations. Treatment of a CHC infection is considered successful if RNA viruses (HCV-RNA) can no longer be detected in the blood between 12 and 24 weeks after treatment is concluded [4, 5, 8]. As initial publications have shown, interferon-free DAA treatments have achieved substantial sustained virological response rates (SVRs) of over 90% in both treatment-naive and treatment-experienced patients with CHC infections. Such treatments are shorter than previous interferon-based therapies, have fewer side effects, and are currently a recommended course of treatment for HCV infections [3, 4].

The purpose of this study is to illustrate the costs for occupational hepatitis C infections based on data of the Institution for Statutory Accident Insurance and Prevention in the Health and Welfare Services (Berufsgenossenschaft für Gesundheitsdienst und Wohlfahrtspflege, BGW) for the period from 2000 to 2014.

## Methods

This study is reported in line with A Consensus German Reporting Standard for Secondary Data Analyses, Version 2 (STROSA) [9].

Secondary data from BGW were used to analyse the costs of occupational HCV infections. BGW’s occupational disease routine data records (BK-DOK) were used as a data source.

### Occupational disease procedure

In Germany, if there is reasonable cause to suspect an occupational disease (OD), physicians are required to submit a report. Reports of suspected ODs may also be submitted by health insurers, employers, representatives of company interests or parties insured with the statutory accident insurance carriers. The statutory accident insurance carriers will follow an investigative procedure to determine whether a disease is an OD as defined by the Occupational Disease Ordinance (*Berufskrankheiten-Verordnung*, BKV) (Section 9, para. 1 & 2 of the German Social Security Code VII). A fully documented connection must be established here between the insured activity and the damaging exposure. The disease must also be fully documented. There must be a probable causal relationship between the exposure and the disease.

In the case of infectious diseases, the exposure is often difficult to identify, which is why it is sufficient to prove that duties have been performed that are associated with an elevated risk of infection. The performance of surgery and contact with blood would be an example of such a hazardous activity with an elevated risk for blood-borne viral diseases. An occupational infectious disease can either be classified as an insurance claim without entitlement to pension disbursement (if the reduced work ability –RWA- is less than 20%), or an entitlement to pension disbursement can be recognised. If there is already a 10% RWA from a previous insurance claim, a new 10% RWA can give rise to a pension disbursement entitlement. In cases where the OD suspicion has not been proven, a rejection is issued.

### Documentation of OD reports

The key features of an OD suspicion report are registered in a standardised fashion in BK-DOK. OD suspicion reports are fundamentally registered as reportable if there are indications of an infection. This applies irrespective of which decision is taken under insurance law in the investigative procedure later. Because the investigative procedure is time-consuming and a decision may not be taken in the year of the OD report, the cases ruled upon in a given reporting year can contain suspicion reports from previous years.

### Analysis

The analysis of the BK-DOK is based on data from insured parties whose HCV infections were reported as suspected ODs between 1996 and 2013. The sample was acquired from the “Insurance Claims” database, which contains not only fundamental personal data but also other details (including sociodemographic features such as sex, year of birth, industry, profession, diagnostic information, year of registration, first and most recent decision, year of decision, OD-triggering circumstances, insurance claim reference number, etc.) The “Compensation Payments” database provided data on expenses incurred on behalf of the insured for drugs and benefits related to medical and occupational rehabilitation.

### Analysis of reduced work ability [RWA]

The RWA classification may change over the course of the disease, depending on the severity of the disease at any given time. The level of impairment and the dates on which it starts and ends are documented for each change in RWA. The analysis of the RWA is based on data from 1996 to 2014 and relates to the first established RWA and the current RWA (as of 31 December 2014). For the presentation of changes in the RWA, CHC patients were sorted according to the current RWA (as of the analysis date) and grouped according to the year of registration of the OD (in five-year groups). Distribution of current RWAs in the respective accident registration periods is presented using percentiles.

Because data on compensation payments have only been collected and processed since 2000, only registrations falling between 1 January 2000 and 31 December 2014 are reflected. The registration titles were grouped and reduced from 100 down to nine categories. In order to represent changes in compensation payments, we have allocated costs to medical rehabilitation (medicines, pharmaceuticals, inpatient and outpatient treatment), occupational rehabilitation (phased return, retraining, etc.) and pension payments (Fig. 2). The results are presented descriptively (absolute and relative frequencies), and the incurred costs are added together over a reporting period of 15 years.

Data on insured parties is provided from within BGW by the Rehabilitation Coordination department. It is derived from the standardised occupational diseases records and is analysed in anonymised form. Data were acquired in accordance with data protection regulations and with the agreement of the BGW Data Protection Officer. The data was analysed using the statistics software SPSS version 23.

## Results

**Fig. 1:**
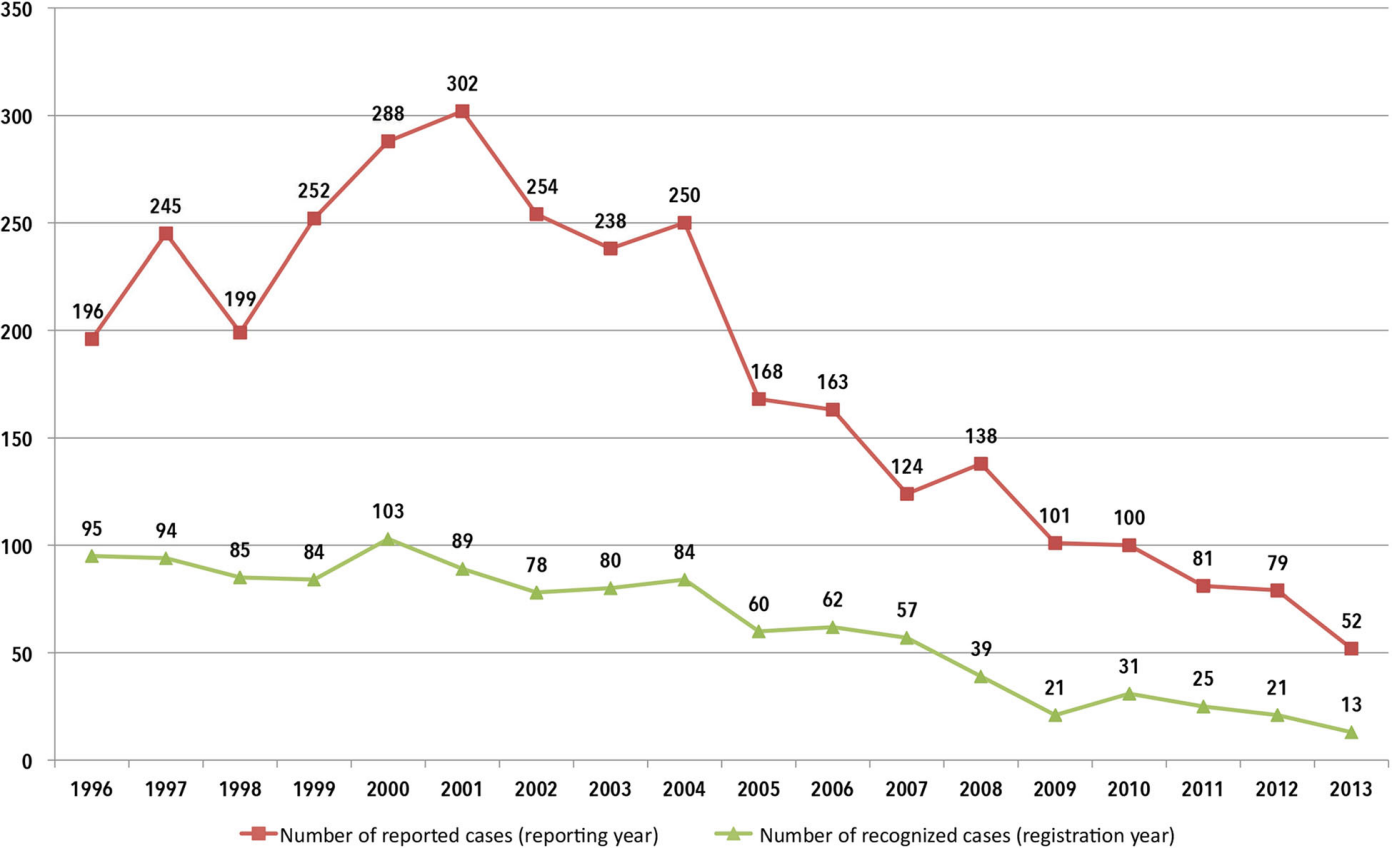
Changes in mandatory reports for suspected hepatitis C and in recognised hepatitis C cases according to registration/reporting year (1996–2013, BGW data)

In 1996 to 2013, a total of 3230 reports of suspected cases of occupational HCV diseases were registered with BGW. In the same period, infections were recognised as ODs for 1121 insured parties. Across the study period, the number of reported and recognised cases declined by 73 and 86% respectively (Fig. 1).

### Description of the sample (OD cases)

OD reports identified 1121 insured parties with an occupational HCV. Mainly affected were female, older than 40 years of age and employed in hospitals. Over 90% of the insured were engaged in a medical or nursing occupation at the time that they developed the disease (Table 1).

**Table 1:** Description of the sample of insured parties with a recognised hepatitis C infection according to sociodemographic characteristics and the degree of reduced work ability (RWA) first established (*n* = 1121)

Characteristics	N	%
Sex, female	838	75
Age (years)^a^		
< 20	17	1
21–40	335	30
41–60	646	58
> 61	123	11
Field of work		
Clinic/hospital	510	46
Doctor’s practice	341	30
Geriatric care/nursing	131	12
Outpatient/social welfare services	79	7
Administration	60	5
Occupation		
Physician	195	17
Nurse	473	42
Medical assistant	266	24
Geriatric care assistant	84	8
Medical personnel	31	3
Administration	36	3
Social worker	19	2
Domestic services	17	1
RWA as %^b^		
No RWA	342	30
RWA <20	3	0.4
RWA 20	493	63.2
RWA 30	127	16.3
RWA 40	64	8.2
RWA 50	35	4.5
RWA 60–80	37	4.7
RWA 100	20	2.6
Total	1121	100

^a^Age at the time of registration of the ODs reports

^b^Values relate to RWA as first established

In the period under analysis, 42 deaths were recorded as the result of an OD. Of those, one person was under 30 years of age, 16 (43%) were between 30 and 60 years of age, while 25 (55%) were over the age of 61. The majority of them (67%) were employed in hospitals, with just 21% working in doctor’s practices and 11% in residential geriatric care or rehabilitation clinics (no table).

### Reduced work ability [RWA]

A total of 70% of insured parties (*n* = 779) had an initial documented RWA in the analysis period from 1996 to 2014. The distribution of the degrees of RWA is shown in Table 1. In 57% of cases, the RWA did not increase during the period of their disease. In just under 40% of cases, there were between two and four changes; only a few had more than five changes. In one case, the RWA changed twelve times.

Of 1121 cases, 75% had no RWA (as of the analysis date of 31 December 2014) (Table 2). These include insured parties who died in the period under analysis (1996 to 2013, *n* = 52). A RWA was established for 779 insured parties over the course of the disease, although a RWA was only in effect for 279 insured parties on the analysis date, showing that 64% of the CHC patients only had a temporary RWA (500 of 779). Breaking down the entire reference period into four intervals shows only minor differences in the RWA grade. Only the 95^th^ percentile and the maximum RWA are higher for cases registered before 2011 than for cases between 2011 and 2013 (40 versus 30% and 100 versus 50%).

**Table 2:** Documented reduced work ability (RWA) as of the analysis date 31 December 2014 grouped by period of occupational disease (OD) registration

RWA as %		Year of OD registration (grouped)									
		1996 to 2000		2001 to 2005		2006 to 2010		2011 to 2013		Total	
		N	%	N	%	N	%	N	%	N	%
	No RWA^a^	357	77	289	74	154	73	42	71	842	75
	010	1	0	0	0	1	0	0	0	2	0
	020	58	13	53	14	26	12	11	19	148	13
	030	15	3	20	5	13	6	4	7	52	5
	040	9	2	7	2	5	2	0	0	21	2
	050	9	2	5	1	7	3	2	3	23	2
	060	4	1	7	2	1	0	0	0	12	1
	070	5	1	4	1	0	0	0	0	9	1
	080	1	0	1	0	1	0	0	0	3	0
	100	2	0	5	1	2	1	0	0	9	1
	Total	461	100	391	100	210	100	59	100	1,121	100
	P 75	0		20		20		20		0	
	P 95	40		40		40		30		40	
	Max	100		100		100		50		100	

^a^Total deaths *n* = 52, of which 42 resulting from occupational disease (1996–2000 *n* = 18; 2001–2005 *n* = 14; 2006–2010 *n* = 8; 2011–2013 *n* = 2); not resulting from occupational disease (1996–2000 *n* = 6; 2001–2005 *n* = 3; 2006–2010 *n* = 1)

### Description of changes in expenses

Table 3 lists the payments and number of postings registered. For 98% of the recognised HCV cases, at least one posting was registered during the period of 15 years (*n* = 1097). Most postings were attributable to outpatient treatments (40%) and pension disbursements (37%). In the period under analysis, compensation payments came to € 87.9 million, of which just under 60% were attributable to pension payments (€ 51,570,830) and around 15% to expenses for pharmaceuticals and other medicines (€ 12,978,318).

**Table 3:** Accounting entry descriptions (grouped) according to number of cases, number of postings and expenses for recognised HCV cases – added together from 2000 to 2014; sorted by percentage share of total costs

Entry descriptions	Number of cases	Number of entries (%)	Expenses in	
			€	%s
Pensions	862	69,585 (37)	51,570,830	59
Pharmaceuticals and other medicines from pharmacies	619	9,897 (5)	12,978,318	14
Injury compensation, care allowances and other treatment costs	586	10,397 (6)	8,461,788	10
Inpatient treatment	543	1,669 (1)	5,460,608	6
Outpatient treatment (including treatment costs by physician)	921	74,376 (40)	4,812,063	6
Diagnosis costs	1,050	14,910 (8)	3,576,946	4
Benefits aimed at enabling involvement in working life (occupational rehabilitation)	49	1,310 (1)	730,423	1
Costs for resources	78	345 (0)	206,087	0
Reports	631	4,492 (2)	60,564	0
Total^a^	5,339^b^	186,981 (100)	87,857,627	100

^a^Does not include combined invoices and payments received for rehabilitation (*n* = 19,445, 9%), ^b^Cases may arise in more than one posting description (e.g., costs for treatments, reports and pharmaceuticals)

**Fig. 2:**
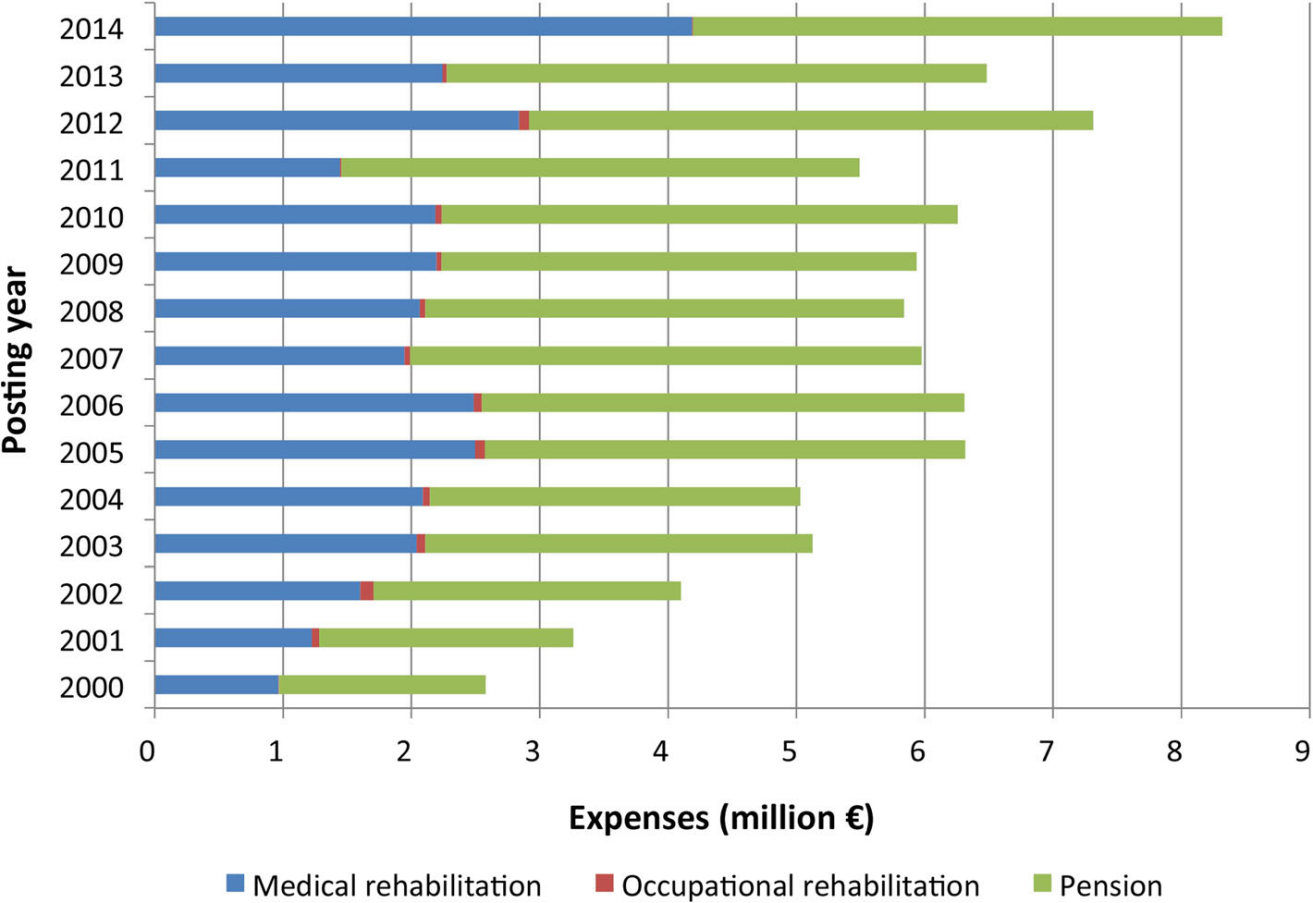
Payments for medical, occupational rehabilitation and pensions for recognised HCV cases; 1097 cases between 2000 and 2014

Compensation payments for medical rehabilitation (pharmaceuticals and other medicines), occupational rehabilitation (phased returns, retraining, etc.) and pensions have developed differently over the 15-year period under review (Fig. 2). Annual expenses rose from € 2.6 million to € 6.3 million between 2000 and 2005, remaining at an annual € 6 million between 2005 and 2010 before then rising to € 7.3 million and € 8.3 million in 2012 and 2014 respectively. In all years except 2014, payments for medical rehabilitation accounted for around one third, while expenses for pensions accounted for around two thirds of the costs. In 2014 on the other hand, expenses for medical rehabilitation accounted for around half of expenditure. Pension payments came to € 1.6 million in 2000 and rose continuously to over € 4 million in 2014. Benefits aimed at enabling involvement in working life (occupational rehabilitation) accounted for less than 1% in all years.

### Drugs for the treatment of hepatitis C

**Fig. 3:**
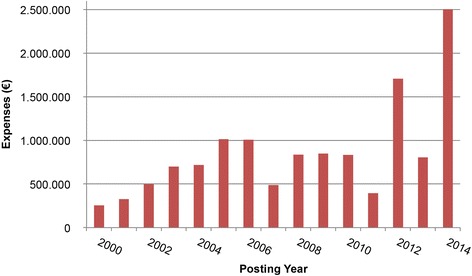
Expenses for drugs for the sample of recognised HCV cases listed by posting year

For the sample of recognised HCV cases, expenses for drugs of € 255,730 in 2000 first rose continuously to around € 1 million and remained at this level until 2010, with the exception of 2007 (Fig. 3). A clear rise in expenses for drugs was recorded for 2012 (to around € 1.7 million) and 2014 (to around € 2.5 million). Relative to the annual costs incurred for the years 2005 to 2010, the increase in costs for drugs was over 70% in 2012 and over 120% in 2014.

## Discussion

Among German healthcare personnel workers, both reported and recognized occupational HCV infections decreased over the study period, yet costs associated with HCV increased. The majority of claims was represented by females aged 40 years and older who were employed as nurses in hospitals. The anti-HCV prevalence in the general population in Germany is estimated to be stable at a low level (0.3%) [10]. The number of reported OD cases among healthcare personnel in Germany declined over the time possibly due to improved blood-borne pathogen handling practices. Guidelines have been issued since 2000 that aim to prevent exposure to blood, for example, from NSI [11]. In the mid-1990s it came to a strong increase in reported ODs, probably in many cases linked to old cases, as a result of increased investigations since the mid-1990s in Germany [12]. In addition, the number of reported and recognized ODs may not reflect the real number of HCV infection cases among healthcare personnel. Needlestick injuries (NSI) are the most frequently reported occupational accidents in healthcare [13]. The results of epidemiological studies indicate that most of HCWs (80%) have been affected by NSI, and many such injuries remain unreported [11].

Despite lower OD HCV prevalence, the costs related to occupational exposure have increased. The costs for CHC are largely defined by the rising expenses for pensions. In 2012 to 2014, however, there was a strong rise in each case in costs for drug therapy. This likely associated with expenses for triple combinations of pegylated interferon, ribavirin and one of the two first-generation protease inhibitors, boceprevir and telaprevir in 2012. The rise in expenses in 2014 is probably attributable to the use of new direct antiviral agents (DAAs, second-generation protease inhibitors) for the treatment of HCV infections.

Initial research results on the new DAAs indicate sustainable SVR rates with shorter treatment periods and a tendency for fewer side effects [4]. DAAs initially involve higher expenses, but may reduce long term treatment costs as reported by Nevens et al. [14]. SVR rates are associated with a reduction in CHC-caused (hepatic and extrahepatic) morbidity and mortality [15, 16]. Functional improvements in the cirrhosis were observed through the SVRs, as were sustained successes in the treatment of extrahepatic complications [4, 8]. CHC remains the most frequent indication for liver transplantation [17]. Preoperative treatment to achieve an SVR at the time of transplantation sustainably reduces the risk of the transplanted organ being infected with the HCV [15, 17]. Early identification of an infection is a key factor in enabling the patient to recover as fully as possible. The treatment of patients in less severe stages of the disease using DAA therapy is costly, but is likely to be successful in preventing future advanced liver diseases that are also associated with high expense [6]. Nevens et al. also observed a considerable reduction in the costs in cases where an SVR was achieved in the mild, less pronounced phases of CHC [14]. In the long term, the high costs of this treatment are likely to pay off by allowing early treatment of CHC infections to reduce morbidity and mortality rates. The case figures presented here do not provide a complete picture of the costs for occupation HCV infections in Germany. The BGW database used here only contains OD reports on employees from non-government institutions. Those restrictions that generally apply for secondary data apply to this register data. The data is not clinical in nature, but rather administrative with limited sociodemographic characteristics present. As in table one reported, nurses are the largest represented demographic group among the healthcare professionals with an HCV infection as OD. This data was not corrected for the total population because it was not possible to get the necessary information, with the same corresponding to the age groups presented. Although this lack of data adjustment is an inherent limitation of our study, a positive feature of this data is that it provides a longitudinal perspective, enabling the claiming of insurance benefits as relevant outcome (e.g., in the form of compensation payments) to be observed over a sufficiently long period of time.

## Conclusions

For hepatitis C infections as an OD, a considerable increase in costs has been observed, while the number of cases has declined heavily. These costs are explained by the increase in pension payments and, since 2012, by a rise in the costs for drugs. The use of DAA treatments is currently fundamentally changing disease management for CHC patients. The high costs of the treatments might be offset by the potentially considerable benefits. The optimum solution is successful therapy at as early a stage in the disease as possible in order to preserve the health-related quality of life of the insured as fully as possible, thereby also preserving their ability to work. The resultant reduction in concomitant hepatic and extrahepatic diseases will potentially result in lower RWA gradings in the long term among the overall insured population. In the long run, maintaining the ability to work would generate overall cost savings for accident insurers and also for the other social security systems.

## Abbreviations

BGW: Berufsgenossenschaft für Gesundheitsdienst und Wohlfahrtspflege
BK-DOK: BGW’s occupational disease routine data records
CHC: Chronic hepatitis C
DAA: Direct anti-viral agent
HCV: Viral hepatitis C
OD: Occupational disease
RWA: Reduced work ability
STROSA: A consensus German reporting standard for secondary data analyses
SVR: Sustained virological response
WHO: World health organisation

## References

[1] Askarian M, Yadollahi M, Kuochak F, Danaei M, Vakili V, Momeni M (2011). Precautions for health care workers to avoid hepatitis B and C virus infection. Int J Occup Environ Med.

[2] Westermann C, Peters C, Lisiak B, Lamberti M, Nienhaus A (2015). The prevalence of hepatitis C among healthcare workers: a systematic review and meta-analysis. Occup Environ Med.

[3] WHO. WHO Guidelines Approved by the Guidelines Review Committee. Geneva: Guidelines for the Screening Care and Treatment of Persons with Chronic Hepatitis C Infection: Updated Version. 2016. (http://www.ncbi.nlm.nih.gov/pubmed/27227200. Accessed 14 Jun 2016). 27227200

[4] Sarrazin C, Berg TP, Buggisch MM, Dollinger H, Hinrichsen H, Hofer DH (2015). Aktuelle Empfehlung zur Therapie der chronischen Hepatitis C S3 guideline hepatitis C addendum. Z Gastroenterol.

[5] WHO. Hepatitis C - Fact sheet Geneva: N°164. 2014. (http://www.who.int/mediacentre/factsheets/fs164/en/. Accessed 4 Feb 2016).

[6] Gordon SC, Pockros PJ, Terrault NA, Hoop RS, Buikema A, Nerenz D (2012). Impact of disease severity on healthcare costs in patients with chronic hepatitis C (CHC) virus infection. Hepatol (Baltimore, Md).

[7] Younossi ZM, Singer ME, Mir HM, Henry L, Hunt S (2014). Impact of interferon free regimens on clinical and cost outcomes for chronic hepatitis C genotype 1 patients. J Hepatol.

[8] Westbrook RH, Dusheiko G (2014). Natural history of hepatitis C. J Hepatol.

[9] Swart E, Bitzer EM, Gothe H, Harling M, Hoffmann F, Horenkamp-Sonntag D (2016). A consensus German reporting standard for secondary data analyses, version 2 (STROSA-STandardisierte BerichtsROutine fur SekundardatenAnalysen). Gesundheitswesen (Bundesverband der Arzte des Offentlichen Gesundheitsdienstes (Germany).

[10] Poethko-Muller C, Zimmermann R, Hamouda O, Faber M, Stark K, Ross RS (2013). Epidemiology of hepatitis a, B, and C among adults in Germany: results of the German health interview and examination survey for adults (DEGS1). Bundesgesundheitsblatt, Gesundheitsforschung, Gesundheitsschutz.

[11] Elseviers MM, Arias-Guillen M, Gorke A, Arens HJ (2014). Sharps injuries amongst healthcare workers: review of incidence, transmissions and costs. J Ren Care.

[12] Dulon ML B, Wendeler D, Nienhaus A (2015). Occupational infectious diseases in healthcare workers 2014. Data from the institution for statutory accident insurance and prevention in the health and welfare services. Zentralbl Arbeitsmed Arbeitsschutz Ergon.

[13] Nienhaus A, Kesavachandran C, Wendeler D, Haamann F, Dulon M (2012). Infectious diseases in healthcare workers - an analysis of the standardised data set of a German compensation board. J Occup Med Toxicol.

[14] Nevens F, Colle I, Michielsen P, Robaeys G, Moreno C, Caekelbergh K (2012). Resource use and cost of hepatitis C-related care. Eur J Gastroenterol Hepatol.

[15] Gonzalez-Grande R, Jimenez-Perez M, Gonzalez Arjona C, Mostazo TJ (2016). New approaches in the treatment of hepatitis C. World J Gastroenterol.

[16] Tada T, Kumada T, Toyoda H, Kiriyama S, Tanikawa M, Hisanaga Y, et al. Viral eradication reduces all-cause mortality in patients with chronic hepatitis C virus infection: a propensity score analysis. Liver Int. 2016;36(6):817–26. 10.1111/liv.13071 26787002

[17] Fagiuoli S, Ravasio R, Luca MG, Baldan A, Pecere S, Vitale A (2015). Management of hepatitis C infection before and after liver transplantation. World J Gastroenterol.

